# Drug resistance profiling of asymptomatic and low-density *Plasmodium falciparum* malaria infections on Ngodhe island, Kenya, using custom dual-indexing next-generation sequencing

**DOI:** 10.1038/s41598-023-38481-3

**Published:** 2023-07-14

**Authors:** Ashley Osborne, Jody E. Phelan, Akira Kaneko, Wataru Kagaya, Chim Chan, Mtakai Ngara, James Kongere, Kiyoshi Kita, Jesse Gitaka, Susana Campino, Taane G. Clark

**Affiliations:** 1 Faculty of Infectious & Tropical Diseases, London School of Hygiene & Tropical Medicine, London, UK; 2 School of Tropical Medicine and Global Health, Nagasaki University, Nagasaki, Japan; 3 Department of Parasitology, Graduate School of Medicine, Osaka Metropolitan University, Osaka, Japan; 4 Department of Microbiology, Tumor and Cell Biology, Karolinska Institutet, Stockholm, Sweden; 5 Centre for Research in Tropical Medicine and Community Development (CRTMCD), Hospital Road Next to Kenyatta National Hospital, Nairobi, Kenya; 6 Directorate of Research and Innovation, Mount Kenya University, Thika, Kenya; 7 Centre for Malaria Elimination, Mount Kenya University, Thika, Kenya; 8 Faculty of Epidemiology and Population Health, London School of Hygiene & Tropical Medicine, London, UK

**Keywords:** Parasite genetics, High-throughput screening

## Abstract

Malaria control initiatives require rapid and reliable methods for the detection and monitoring of molecular markers associated with antimalarial drug resistance in *Plasmodium falciparum* parasites. Ngodhe island, Kenya, presents a unique malaria profile, with lower *P. falciparum* incidence rates than the surrounding region, and a high proportion of sub-microscopic and low-density infections. Here, using custom dual-indexing and Illumina next generation sequencing, we generate resistance profiles on seventy asymptomatic and low-density *P. falciparum infections* from a mass drug administration program implemented on Ngodhe island between 2015 and 2016. Our assay encompasses established molecular markers on the *Pfcrt*,* Pfmdr1*,* Pfdhps*,* Pfdhfr*, and *Pfk13* genes. Resistance markers for sulfadoxine-pyrimethamine were identified at high frequencies, including a quintuple mutant haplotype (*Pfdhfr/Pfdhps*: N51I, C59R, S108N/A437G, K540E) identified in 62.2% of isolates. The *Pfdhps* K540E biomarker, used to inform decision making for intermittent preventative treatment in pregnancy, was identified in 79.2% of isolates. Several variants on *Pfmdr1*, associated with reduced susceptibility to quinolones and lumefantrine, were also identified (Y184F 47.1%; D1246Y 16.0%; N86 98%). Overall, we have presented a low-cost and extendable approach that can provide timely genetic profiles to inform clinical and surveillance activities, especially in settings with abundant low-density infections, seeking malaria elimination.

## Introduction

Despite rapid progress in disease control and elimination efforts between the years 2000 and 2015, malaria continues to be a major public health burden, particularly in low- and middle-income countries. Caused by parasite species within the *Plasmodium* genus, malaria was responsible for 247 million cases worldwide and contributed to 619,000 deaths in 2021 alone^1^. In Sub-Saharan Africa, *P. falciparum* malaria carries the heaviest burden, accounting for an estimated 95% of all cases and 96% of all deaths, with about 80% of mortality occurring among children less than 5 years of age. Progress towards reduction in disease incidence, and ultimately elimination, has been hampered by the emergence and spread of antimalarial resistance mechanisms in *Plasmodium* parasites, insecticide resistance in mosquito vectors, and ecological threats associated with global climate change. These setbacks have been further exacerbated by malaria control programme and supply chain interruptions caused by the COVID-19 pandemic, as well as shortcomings in global funding for malaria elimination in the wake of the pandemic^2,3^.

Malaria incidence rates and transmission risk have declined across much of Kenya, with national malaria prevalence estimated to be below 10%, although true numbers of cases remain unknown as asymptomatic individuals are far less likely to seek out diagnosis and treatment^4^. Despite an overall reduction in incidence, lower elevation regions in Kenya, including those along the Indian Ocean coast and Lake Victoria, still experience a high malaria burden^5^. Lake Victoria remains a region of intense transmission due to various environmental and geopolitical factors that have made targeted vector control and tailored malaria control programmes difficult to implement region-wide^6–8^. During peak transmission periods, determined by the two rainy seasons in March to May and October to December, *P. falciparum* prevalence in the Lake Victoria basin can reach up to 40% in individuals aged between 2 and 10 years^5,7,9^. Overall prevalence, however, can vary drastically amongst the islands and mainland populations^7^. In Homa Bay County, Suba District (population size: North 124,938; South 122,383) and the large island of Mfangano (population size 26,000) generally have the highest parasite prevalence rates, sometimes even exceeding 40%, while the smaller islands, including Ngodhe island (population size 600–1000), are generally associated with lower prevalence rates and asymptomatic, sub-microscopic, infections^5,7,10^.

In 2015, the World Health Organization (WHO) recommended the implementation of mass drug administration (MDA) in low transmission regions, including inhabited islands^11^. Despite its close geographical proximity to regions of intense malaria transmission, Ngodhe island maintains overall low levels of transmission, as well as high proportions of asymptomatic and sub-microscopic infections. This unique transmission profile on Ngodhe island led to the rationalisation of using an MDA strategy in 2015 and 2016 to assess the efficacy of an MDA in reducing malaria on islands with high and heterogeneous transmission^7,12^. A high compliance MDA with artemisinin-based combination therapies (ACTs) and a single low dose of primaquine, with approximately 90% participation, led to an initial decrease in parasite prevalence from 3% to 0% by microscopy and 10% to 2% by PCR. Despite this initial drastic decrease, prevalence rebounded to baseline levels within six months, and in 2017 was recorded to be 4.6% and 16.0%, respectively^7^. The island’s proximity to regions with high malaria transmission and an inability to prevent parasite importation likely prevented sustainable elimination conditions from being achieved^7^.

Difficulties in malaria control, and the recent emergence of parasites with reduced susceptibility to artemisinin, a component of ACTs, in Uganda and Rwanda, have highlighted the need for rapid and reliable methods for detecting and monitoring molecular markers associated with drug resistance in *P. falciparum*^13,14^. ACTs are the first-line treatment for uncomplicated malaria and last-line of defence following the widespread global emergence of chloroquine and sulfadoxine-pyrimethamine (SP) resistance amongst parasite populations^15–18^. Despite widespread resistance, SP is still used as an intermittent preventive treatment during pregnancy (IPTp), in accordance with WHO guidelines^2^. Although chloroquine has been discontinued, molecular markers for chloroquine-resistance persist in parasite populations across Africa^17^. There is recent evidence of chloroquine-sensitivity returning in countries that adopted more rapid changes in malaria treatment policies, suggesting chloroquine could be reintroduced in the future^19–23^. There are well categorised and studied biomarkers for antimalarial drug resistance, namely single nucleotide polymorphisms (SNPs), on *P. falciparum* genes *Pfcrt, Pfmdr1*, *Pfdhfr*, and *Pfdhps* that confer drug resistance to chloroquine, SP, and ACT partner drugs, as well as SNPs on *Pfk13* associated with delayed parasite clearance to ACTs^16,24–27^.

Advancements in low-cost sequencing-based approaches that target molecular markers for drug resistance (e.g. amplicon sequencing) offer a new high throughput method of parasite surveillance, although have not been utilised to look specifically at low-density or asymptomatic infections^28–30^. Due to the high proportion of sub-microscopic and asymptomatic *P. falciparum* infections on Ngodhe island, no genetic information is available for the island’s parasite population, including drug resistance frequencies^7^. Here we demonstrate the use of custom dual-indexing and Illumina next generation sequencing-technology, to generate a resistance profile of the *P. falciparum* parasites on Ngodhe island from predominantly asymptomatic and low-density, infections collected during MDA activities between 2015 and 2016. Our analysis was able to quantify molecular markers of resistance on the *Pfcrt*, *Pfmdr1*, *Pfdhps*, *Pfdhfr*, and *Pfk13* genes, establishing this method as a viable means of malaria surveillance suitable for regions with typical sub-microscopic infections.

## Results

### Illumina amplicon sequencing and coverage

**Figure 1:**
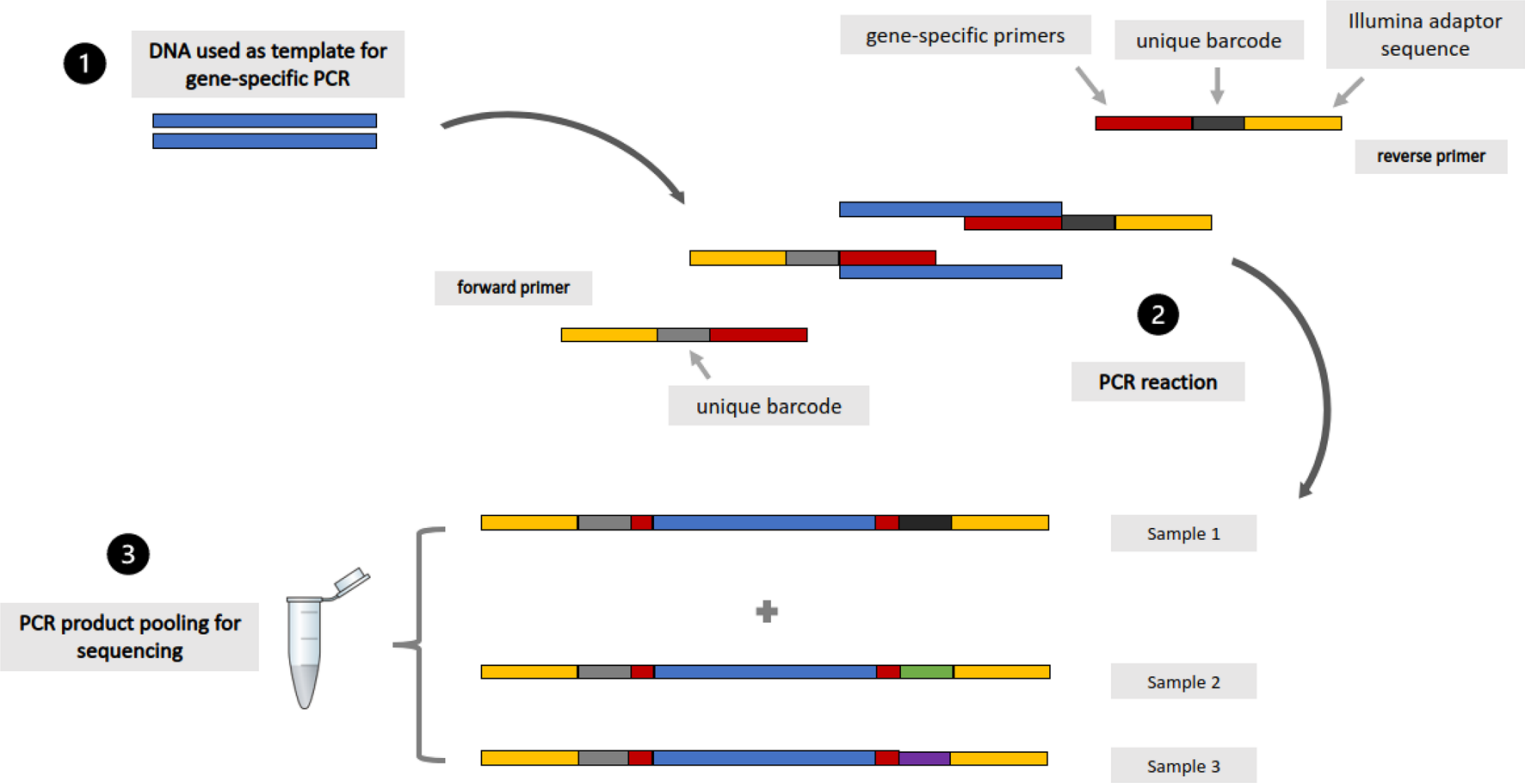
Primer and fragment design using custom dual indices for amplicon generation prior to Illumina sequencing.

In years 2015 and 2016, a high compliance (> 90% participation) MDA was carried out on Ngodhe island. Of the 201 PCR-positive dried blood spot (DBS) samples collected during the 2015/2016 MDA campaign on the island, 102 (53.7%) samples were deemed suitable for sequencing based on low DNA concentration measurements (Qubit HS DNA concentration > 0.4 ng/ul). From the 102 *P. falciparum* samples, 70 (68.7%) were successfully sequenced on an Illumina platform to cover a total of nine 500 base pair amplicons that encompassed five genes (*Pfk13, Pfcrt, Pfmdr1, Pfdhps*, and *Pfdhfr*) (Table S1; Fig. 1). The 70 sequenced samples were sourced from 49 (70.0%) sub-microscopic infections, 12 (17.1%) low-parasitaemia (< 1000 parasites/µl blood) and 9 (12.8%) moderate/high-parasitaemia infections (> 1000 parasites/µl blood). Rapid diagnostic test (RDT) data was available for 24 of the sequenced samples, 5 with negative and 19 positive RDT results. Of the 5 samples that were negative by RDT but positive by PCR, 4 were also negative by microscopy. Individuals were classified as asymptomatic with an absence of fever or other acute symptoms^4^.

**Figure 2:**
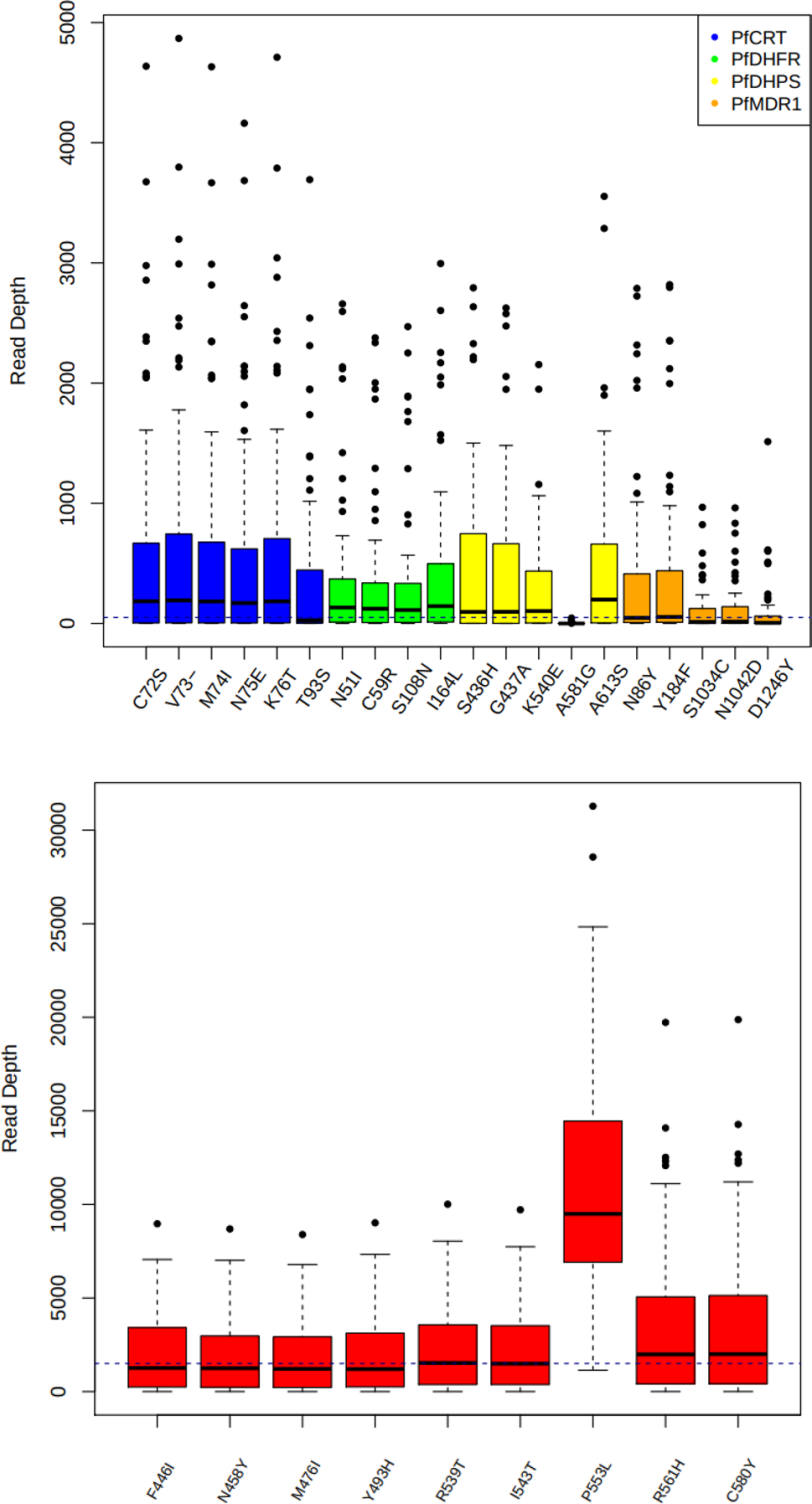
Read depth across targeted mutations within amplicons covering five drug resistance genes; (top) non-*Pfk13*, with a trendline of 50-fold coverage; (bottom) *Pfk13* gene, with a trendline at 1500-fold coverage.

The median depth of read coverage achieved via amplicon sequencing overall was high (range of medians: 69- to 1760-fold; Table S1), with *Pfk13* (1760-fold) having noticeably higher average coverage than *Pfcrt, Pfmdr1, Pfdhps*, and *Pfdhfr* (69- to 155-fold) (Table S1; Fig. 2). In general, regional coverage of 20-fold or higher is considered adequate for genetic studies. Low coverage of codon 581 on *Pfdhps* was linked to a drop in coverage in the middle of the amplicon shared with codons 540 and 613. High coverage of codon 553 on *Pfk13* (median 9493-fold) was due to primer overlap from adjacent amplicons.

### SNP frequencies on drug resistance-associated genes using Illumina sequencing

Across the 70 samples sequenced using the Illumina platform, only SNPs with a minimum read depth > tenfold on *Pfcrt*, *Pfmdr1*, *Pfk13, Pfdhps*, and *Pfdhfr* were included in this analysis to ensure accuracy in SNP reporting. For context and comparison, variant frequencies across these genes were compared to previously categorised frequencies from parasites on Mfangano island, Lake Victoria, as well as East and West African isolates available via the Pf3k database (Table 1)^10^.

**Table 1:** Minor allele frequencies (MAF) of variants identified within the *P. falciparum* parasites on Ngodhe island and their respective frequencies across East African and West African parasite populations (minimum read depth > tenfold).

Gene	SNP	Ngodhe island			Mfangano island			East Africa^1^	West Africa^2^
		MAF	95% CI	*n*	MAF	95% CI	*n*	MAF (*n* = 228)	MAF (*n* = 167)
*Pfcrt*	**M74I**	**6.5**	**2.2–18**	**46**	**0**	**0–12**	**27**	**0**	**0**
	**N75E**	**4.4**	**1.5–15**	**45**	**0**	**0–12**	**27**	**0**	**0**
	**K76T**	**4.4**	**1.5–15**	**45**	**7.4**	**2.1–23**	**27**	**17.5**	**13.0**
	H97Y	0	0**–**7.9	45	0	0**–**12	27	0	0
*Pfmdr1*	F61Y	3.5	0.97**–**12	57	0	0**–**11	30	0	0
	**N86Y**	**2.0**	**0.35–10**	**50**	**0**	**0–11**	**30**	**15.7**	**24.4**
	**Y184F**	**47.1**	**34–60**	**51**	**31.7**	**0–11**	**30**	**39.0**	**15.5**
	T199S	13.5	6.7**–**25	52	3.1	0.55**–**16	32	0.01	0
	F938Y	3.0	0.83**–**10	66	0	0**–**11	31	8.3	22.2
	F1072Y	2.0	0.37**–**11	48	0	0**–**10	35	0	0
	**D1246Y**	**16.0**	**7.1–33**	**31**	**2.9**	**0.51–15**	**35**	**12.9**	**31.2**
*Pfk13*	A578S	3.1	0.83**–**11	65	0	0**–**10	35	1.1	0
	F451Y	4.5	1.6**–**13	66	0	0**–**10	35	0	0
*Pfdhfr*	**N51I**	**77.0**	**62–85**	**53**	**100**	**90–100**	**34**	**91.4**	**92.5**
	**C59R**	**76.9**	**64–86**	**52**	**91.2**	**77–97**	**34**	**86.4**	**93.0**
	**S108N**	**80.8**	**68–89**	**52**	**100**	**90–100**	**36**	**99.4**	**93.0**
	**I164L**	**5.8**	**2.0–16**	**52**	**6.9**	**2.9–22**	**36**	**1.5**	**0**
*Pfdhps*	S436H	11.9	5.2**–**25	42	22.9	12**–**39	35	0	0
	**G437A**	**100**	**92–100**	**42**	**100.0**	**90–100**	**35**	**85.3**	**70.0**
	**K540E**	**79.2**	**66–88**	**48**	**95.7**	**85–99**	**35**	**84.4**	**30.0**

^1^East Africa: Kenya, Tanzania, and Uganda.

^2^West Africa: The Gambia and Mauritania.

bold - mutations with known resistance associations.

There were 3 nonsynonymous mutations observed on the *Pfcrt* gene, resulting in amino acid changes M74I, N75E, and K76T. These variants are well documented to be associated with resistance to chloroquine. The *Pfcrt* N74I polymorphism occurred in 3 out of 46 samples while the N75E and K76T polymorphisms were observed in 2 out of 45 samples. The *Pfcrt* K76T biomarker, the primary marker for chloroquine resistance, was observed in 4.4% of the Ngodhe samples, compared to 7.4% of isolates from Mfangano island in 2020 and 17.5% from wider East African populations^10^. The *Pfcrt* C72S and V73V variants were not observed. Analysis of haplotype frequencies on *Pfcrt* for samples with a read depth > tenfold at every position (codons 72, 73, 74, 75, and 76) identified the distributions of mutants and wild type parasites in the Ngodhe parasite population (*n* = 45) (Table 2). The wild-type parasites, CVMNK, made up 95.5% of the population while the triple mutant, CV**IET** (mutations underlined), accounted for 4.5% of the population.

**Table 2:** Drug resistance haplotype frequencies on *Pfcrt and Pfmdr1* identified within the Ngodhe island *P. falciparum* parasite population (minimum read depth > tenfold).

No. of samples	*Pfcrt* haplotype					Freq. (%)	
	C72S	V73	M741	N75E	K76T		
43*	C	V	M	N	K	95.5	
2	C	V	**I**	**E**	**T**	4.5	

No. of samples	*Pfmdr1* haplotype					Freq. (%)	
	N86Y	Y184F	F938Y	S1034C	N1042D	D1246Y	
14*	N	Y	F	S	N	D	45.2
12	N	**F**	F	S	N	D	38.7
3	N	Y	F	S	N	**Y**	9.7
2	N	**F**	F	S	N	**Y**	6.4

Amino acid changes in bold.

*Wild type (WT).

On the *Pfmdr1* gene, 7 nonsynonymous SNPs were identified, 3 of which are reported to cause amino acid changes resulting in altered susceptibility to chloroquine and the ACT partner drug lumefantrine (N86Y, Y184F and D1246Y). The *Pfmdr1* N86Y variant, where the wild-type (WT) allele is believed to be selected for by artemether–lumefantrine usage, was identified in 1 out of 50 samples (2.0%), appearing at similar frequencies to Mfangano island isolates (0%)^10^. The D1246Y mutation was observed in 5 out of 31 samples (16.0%), appearing at a higher frequency compared to Mfangano isolates (2.9%). Frequencies in East African isolates were higher for *Pfmdr1* N86Y, 15.7%, while the N1246Y biomarker appeared at a similar frequency to Ngodhe isolates (12.9%). The *Pfmdr1* Y184F biomarker was observed in 47.1% of samples (24 out of 51 isolates). This frequency was slightly higher than frequencies observed in Mfangano island (31.7%), and East African isolates (39.0%)^10^. The *Pfmdr1* S1034C and N1042D variants were not observed in any of the 70 samples with read depth more than tenfold at this position.

Haplotype analysis of *Pfmdr1* identified the frequencies of single and double mutants within the Ngodhe parasite populations based on codon positions 86, 184, 938, 1034,1042, and 1246 (*n* = 31) (Table 2). Wild-type parasites, *Pfmdr1* NYFSND, made up the largest fraction of the population, accounting for 45.2% of isolates, while the single mutant, N**F**FSND, made up 38.7%. The single mutant, *Pfmdr1* NYFSN**Y**, accounted for 9.7% of the population while the double mutant, N**F**FSN**Y**, constituted the remaining isolates at 6.4%. No drug resistance-associated polymorphisms were recorded on *Pfk13.* The *Pfk13* variant A578S was recorded but is not thought to confer reduced susceptibility to artemisinin or any other antimalarial drugs.

There were 4 nonsynonymous SNPs identified on *Pfdhfr*, all of which resulting in amino acid changes documented to confer reduced susceptibility to pyrimethamine (N51I, C59R, S108N, and I164L). N51I, C59R, AND S108N changes were seen at similar frequencies amongst the Ngodhe isolates, with prevalence observed at 77.0%, 76.9%, and 80.8%, respectively (Table 1). Isolates from Mfangano island and East Africa populations were observed to have all 3 biomarkers at higher frequencies (> 87%) than Ngodhe island^10^. The I164L variant was observed in 5.2% of Ngodhe isolates, similar to frequencies to Mfangano isolates (6.9%), and higher than isolates from East Africa (1.5%) and West Africa (0%).

On *Pfdhps*, 3 nonsynonymous SNPs were observed, with 2 known to result in amino acid changes associated with resistance to sulphadoxine, A437G and K540E. The K540E mutant was observed in 79.2% of isolates and is generally used as a proxy measure for the presence of all 5 key mutations for resistance to SP (N51I, C59R, S108N, I164L, A437G, and K540E). The A437G mutation was observed to be fixed at 100% in samples from Ngodhe and Mfangano islands, compared to 84.4% in samples from wider East African populations.

Analysis of the Ngodhe parasite *Pfdhfr/Pfdhps* haplotype frequencies, for samples with a read depth more than tenfold at every nucleotide position, included *Pfdhfr* codons 51, 59, 108, and 164, as well as *Pfdhps* codons 437, 540, 581 (*n* = 37) (Table 3). The quintuple mutant haplotype, **IRN**I**GE**A, was observed in a majority of screened Ngodhe isolates, accounting for 62.2% of the population, as well as a sextuple mutant haplotype, **IRNLGE**A, which was identified in 5.4% of isolates. The single mutant haplotype, NCSI**G**KA, was observed at the second highest frequency, 13.5%, while the wild-type haplotype, NCSIAKA, was not observed in any isolates. Double, triple, and quadruple mutants accounted for the remainder of the population, occurring at frequencies ranging from 2.7% to 5.4%.

**Table 3:** Drug resistance haplotype frequencies on *Pfdhfr*/*Pfdhps* identified within the Ngodhe island *P. falciparum* parasite population (minimum read depth > tenfold).

No. of samples	*Pfdhfr/Pfdhps*							Freq. (%)
	N51I	C59R	S108N	I164L	A437G	K540E	A581G	
23	**I**	**R**	**N**	**I**	**G**	**E**	A	62.2
5	N	C	S	I	**G**	K	A	13.5
2	**I**	**R**	**N**	I	**G**	K	A	5.4
2	**I**	**R**	**N**	**L**	**G**	**E**	A	5.4
2	N	C	S	I	**G**	**E**	A	5.4
1	**I**	C	**N**	I	**G**	K	A	2.7
1	**I**	C	**N**	I	**G**	**E**	A	2.7
1	N	**R**	**N**	I	**G**	**E**	A	2.7

Amino acid changes in bold.

## Discussion

Advancements in targeted low-cost sequencing approaches provide a viable means for monitoring the emergence and spread of characterised drug resistance-associated polymorphisms in both asymptomatic and low-density *P. falciparum* infections^28,31^. Asymptomatic infections tend to be under-represented in large-scale genetic analyses, despite accounting for most infections worldwide^32^. This is often due to the difficulties associated with extracting sufficient DNA needed to perform genomic characterisation, such as whole genome sequencing. Additionally, asymptomatic individuals do not tend to seek treatment and go undiagnosed. As countries make progress towards their malaria elimination goals, sub-microscopic and asymptomatic cases will remain the main reservoirs for disease^1^. To ensure accurate and informed disease control policies, molecular surveillance methods need to be sufficiently sensitive to detect regions of interest, such as drug resistance biomarkers, in these types of infections.

To assist in the generation of higher resolution drug resistance profiles of malaria parasites, we demonstrate the use of custom dual-indexing amplicon sequencing technology to identify molecular markers of resistance on *Pfcrt*, *Pfmdr1*, *Pfdhps*, *Pfdhfr*, and *Pfk13* genes from asymptomatic and low-density *P. falciparum* infections on Ngodhe island in Lake Victoria, Kenya. Ngodhe island has a unique transmission profile compared to surrounding inhabited islands and mainland communities located within western Kenya and Lake Victoria. In a region of intense transmission, asymptomatic and sub-microscopic infections make up most cases detected on Ngodhe island, which also has an overall lower *P. falciparum* incidence^7^. Given these distinctive characteristics, assessing resistance biomarkers within Ngodhe island’s *P. falciparum* population for the first time provided an invaluable baseline for drug resistance monitoring, as well as provided data that can be utilised to better inform policy making decisions concerning malaria control programmes within the region.

To preserve the efficacy of antimalarial drugs, monotherapy treatments (e.g. chloroquine) have been phased out for the treatment of uncomplicated malaria and replaced by ACTs in much of world, including Kenya^5,33^. Despite its discontinued use, chloroquine can still occasionally be found at local pharmacies in parts of Africa as a general treatment for fevers^1^. Monitoring chloroquine resistance mutations in parasite populations can provide insight into the presence of any ongoing drug selection pressure, which could be due to ongoing sales of chloroquine, as well as the return of chloroquine sensitivity, as seen in Malawi, following the complete removal of chloroquine from circulation^23^. The K76T mutation on *Pfcrt*, often used as the main molecular marker for chloroquine resistance, was observed to be at low frequencies in Ngodhe island parasites, with the wild-type K76 allele identified in most of the isolates screened through this study. The same was found to be true for other resistance conferring mutations on *Pfcrt*, with only wild-type alleles observed at codons 72 and 73, and high frequencies of wild-type alleles at codons 73 and 74. These results support the hypothesis that removal of chloroquine drug selection pressure may promote the return of chloroquine sensitive wild-type parasites^19–22^. Additionally, it has been documented that treatment with artemether-lumefantrine, extensively used in East Africa, selects for the chloroquine-susceptible *Pfcrt* K76 allele, suggesting that the K76T mutation may be a drug-specific contributor to enhanced *P. falciparum* susceptibility to lumefantrine^34,35^.

In accordance with WHO guidelines, SP continues to be used as IPTp in pregnant women but is not used as a first-line treatment for uncomplicated malaria^2^. Nonsynonymous polymorphisms on *Pfdhfr* and *Pfdhps* are associated with resistance to pyrimethamine and sulphadoxine, respectively. The degree of resistance to SP increases with each subsequent mutation on these genes, which has led to the emergence of quadruple, quintuple, and, more recently, sextuple mutations^25,36^. Resistance markers for SP were identified at high frequencies in Ngodhe island isolates, likely due to the continued drug selection pressure within the parasite population. The *Pfdhfr/Pfdhps* quintuple mutant haplotype, encompassing the N51I, C59R, S108N, A437G and K540E mutations, was observed to be the most prominent haplotype, accounting for 62.2% of the screened population. Additionally, a sextuple mutant haplotype, including *Pfdhfr/Pfdhps* variants N51I, C59R, S108N, I164L, A437G and K540E, was identified in two isolates. Given the high frequencies of these SP resistance-associated biomarkers and continued circulation of SP in local parasite populations, there exists the possibility of relatively low fitness costs associated with maintaining these mutations.

The *Pfdhps* K540E variant is used as a proxy measure for the presence of all 5 key mutations associated with SP resistance on *Pfdhfr* and *Pfdhps*. However, following haplotype analysis, the K540E mutation was also observed in double and quadruple mutants at frequencies ranging from 2.7 to 5.4%^37,38^. In addition to being a proxy measure for SP resistance, the K540E mutation is also used to inform decision making by the WHO surrounding IPTp guidance, with IPTp implementation only recommended in regions where K540E prevalence is < 50%^39^. The K540E marker was identified in 79.2% of the Ngodhe island isolates, suggesting there may be reduced efficacy of IPTp-SP therapies within the region. The high frequency of this mutation highlights the need for continued monitoring of malaria infections in pregnant women to prevent adverse malaria-related outcomes, as well as the need for more safe and effective malaria drugs for use in pregnant women^40^.

There were no mutations observed on *Pfk13* believed to confer a reduced susceptibility to artemisinin, however there were a handful of variants identified on *Pfmdr1* associated with reduced susceptibility to the ACT partner drug lumefantrine. To prevent artemisinin resistance, ACTs combine artemisinin, a fast acting and highly effective antimalarial drug, with a partner drug that has a longer half-life to clear any remaining parasites^41,42^. Resistance to ACT partner drugs poses a threat to maintaining the efficacy of these combination therapies and the protection they provide against the emergence of artemisinin resistance. Artemether-lumefantrine is the ACT extensively used across much of Africa, including Kenya where it was introduced in 2006^27^. The *Pfmdr1* Y184F and D1246Y variants, identified in 47.1% and 16% of isolates respectively, are associated with varying degrees of reduced susceptibility to lumefantrine and quinolines. Recent studies have found evidence that the Y184F mutant allele is not the primary determinant of resistance to lumefantrine but that there may be a genetic correlation between Y184F and the acquisition of a drug-resistance phenotype^26^. The N86Y variant, identified in 2% of isolates, is believed to be associated with changes to susceptibility of chloroquine and amodiaquine. The N86 wild-type codon, conversely, may confer a reduced susceptibility to lumefantrine and was identified in 98% of Ngodhe isolates^43^.

There were several limitations with this study, especially related to the use of field isolates. The samples were collected as part of an MDA that occurred throughout 2015 and 2016. Due to the nature of malaria parasites and their ability to adapt quickly to selective pressures, drug resistance polymorphism prevalence may differ from this sample set and current day prevalence. However, there was previously no data available for this region and this study aimed to set a baseline for future studies, as well as demonstrate amplicon sequencing as a viable method for screening low-density malaria infections. Additionally, we would anticipate differences over time in the frequencies of molecular markers for drug resistance regardless of the 2015/2016 MDA as *P. falciparum* populations are always adapting to changes in drug pressures within the environment. Drug resistance linked to copy number variation, particularly relevant for *Pfmdr1*, was not addressed by this technique and leaves room for future work to address and supplement this methodology. However, amplification of *Pfmdr1* is considered rare in East African parasite populations and is not believed to play a significant role in drug resistance in this region^44^. Finally, only 102 (of the 201) PCR-positive isolates were sequenced. The sequenced isolates had the highest DNA concentrations, and it is possible this could have led to some sampling bias and mutations could have been missed.

Progress towards global malaria eradication over the past few years has been hampered by the emergence and spread of resistance to antimalarial drugs and has highlighted the need for rapid and reliable methods to detect and monitor molecular markers of drug resistance. Here we applied amplicon-based approaches aimed at targeting known and putative drug resistance markers in established loci on *Pfcrt*, *Pfmdr1*, *Pfdhps*, *Pfdhfr*, and *Pfk13* to demonstrate the effectiveness of sequencing-based high throughput surveillance in a low resource setting on asymptomatic, low-density, *P. falciparum* infections. The cost of sequencing has been prohibitive to the implementation of sequencing-based surveillance methods in low-resource settings. However, thanks to the development and application of dual-indexing technology, alongside targeted short-read sequencing, amplicon sequencing presents an affordable alternative to whole genome sequencing, as well as offers the potential for cross-platform capability, including both Illumina and Oxford Nanopore Technology sequencing platforms. In addition to more inclusive pricing (currently < USD 0.5 per amplicon), amplicon sequencing is easily expandable to include a wider range of targets across the Plasmodium genome, as well as targets within other species, suggesting the possibility of an integrated host–pathogen-vector surveillance system.

## Methods

### Study site selection

In 2015 and 2016, a high compliance (> 90% participation) MDA was carried out on Ngodhe island in Lake Victoria (population size 600–1000)^7^. Throughout the duration of the MDA, a total of 3,167 dry blood spot (DBS) samples were collected, including: 184 pre-MDA, 458 on day 0, 391 on day 2, 372 on day 7, 459 on day 35, 387 on day 42, 462 on day 120, and 454 on day 180. Samples were collected from all MDA participants, which encompassed > 90% of the island’s total population, irrespective of clinical symptoms, gender, or age. Of the 3,167 samples, 201 samples were positive by PCR. PCR positive individuals were treated with Artequick® (artemisinin and piperaquine combination) and primaquine. Rapid diagnostic test data was available for samples collected during the follow-up phase of the MDA, which began on day 120. Of 201 PCR-positive DBS samples collected during the 2015/2016 MDA campaign on Ngodhe island, 102 (53.7%) samples were selected for sequencing based on Qubit HS DNA concentration measurements (DNA concentration > 0.4 ng/ul).

Malaria species identification and parasite density assessment were carried out using microscopy, following WHO guidelines, at the Nagasaki University research station in Mbita, Kenya by trained microscopists and confirmed at Osaka Metropolitan University and LSHTM Malaria Reference Laboratory using established nested PCR assays^45,46^. Ngodhe island is occupied by the Luo ethnic group, migrant fishermen and families. The island is less than 1 km^2^ in size and accessible only by small boat; located 3 km north of Rusinga island and the mainland town of Mbita. The use of long-lasting insecticide treated bed nets (LLINs) on the island increased between 2012 and 2015. This uptake was due to initiatives introduced by the Kenyan Ministry of Health to provide free LLINs for households and pregnant women. This increase in LLINs usage saw a decrease in malaria prevalence on the island during the 2012–2015 time-period. Indoor residual spraying (IRS) was not implemented on Ngodhe island until 2018.

### Primer design

Primers used in this study were designed to investigate polymorphisms in *Pfcrt* (codons 72–76), *Pfmdr1* (codons 86, 184, 1034, 1042, and 1246), *pfdhfr* (codons 16, 51, 59, 108, and 164), *pfdhps* (codons 431, 436, 437, 540, 581, and 613), and the propeller domain of *pfK13* by amplifying fragments, or amplicons, of between 500 and 600 base pairs (bp) (Table S1). Primers were designed to contain custom 6-nucleotide indices based on previously described and published methodologies by Nag et al.^27^. In this study, the *Pfcrt* primers were further optimised from those published by *Nag *et al*.* for use in Pf3D7, and field isolates, while the *Pfmdr1* primer set was expanded to include the codon at position 184.

### DNA extraction and sample preparation

DNA from isolates collected in years 2015 and 2016 was extracted in 2021 from dried-blood spots (DBS) preserved on filter papers. Filter papers were stored at 4 °C from 2015/2016 until blood extraction in 2021. A cold chain was not maintained during sample shipment from Kenya to the London School of Hygiene and Tropical Medicine. For DNA extraction, half of a DBS was used. DNA extraction was performed using the Qiagen QiaSymphony Automated Nucleic Acid Extraction Facility and the Qiagen QIAsymphony DSP DNA kit. Following extraction, DNA was amplified using an established selective whole genome amplification (SWGA) primer set and protocols^31,47^.

### PCR reactions and programmes

Simplex PCRs, carried out for *Pfcrt*, were performed using a mastermix containing 5 µl of Q5 Reaction Buffer (New England BioLabs), 0.5 µl of dNTPs (1n nM stocks, New England BioLabs), 0.25 µl Q5 Hot Start High-Fidelity DNA Polymerase (New England BioLabs), and 15.75 µl Milli-Q water (Merck). For each reaction, a total of 1.25 µl of forward and 1.25 µl of reverse primer (10 pmol/µl stocks) were used with 1 µl of DNA for a total reaction volume of 25 µl. Multiplex PCRs (combinations described in Supplementary Table 2) were performed using a mastermix containing 5 µl of Q5 Reaction Buffer (New England BioLabs), 0.5 µl of dNTPs (1n nM stocks, New England BioLabs), 0.25 µl Q5 Hot Start High-Fidelity DNA Polymerase (New England BioLabs), and 15.8 µl Milli-Q water (Merck). For each multiplex reaction, 0.6 µl of both forward primers, for a total of 1.2 µl of forward primer, and 0.6 µl of each reverse primer, for a total of 1.2 µl of reverse primer, were used with 1 µl of DNA for a total reaction volume of 25 µl. The reactions were carried out in a thermocycler consisting of the following steps: Heat activation for 15 min at 72 °C, 30 cycles of denaturation for 20 s at 95 °C, annealing for 2 min at 55 °C, elongation for 2 min at 72 °C, and a final elongation for 10 min at 72 °C, followed by a hold at 10 °C.

### Amplicon purification and pooling

Indices for each amplicon target were conserved according to the sample identifier. Samples not containing shared index combinations were pooled (a maximum of 110 amplicons per pool was achieved) for purification (Fig. 1). Pooled samples were purified prior to sequencing using bead purification (KAPA), according to the manufacturer's instructions, using a ratio of 1:0.70 of product to bead volume to select for 400 to 500 bp segments of DNA. DNA concentration was measured using Qubit dsDNA HS (Invitrogen) and standardised to a final concentration of 20 ng per 25 µl.

### Illumina sequencing and bioinformatics

*P. falciparum* isolates collected in 2015/2016 (n = 102) were sequenced using the Illumina MiSeq platform with 300 bp paired end kits at Genewiz (GENEWIZ Germany GmbH). The sequencing reads from pooled samples were demultiplexed, divided into separate files based on their unique indices, using an in-house python script to generate individual FASTQ files necessary for downstream analysis. Following demultiplexing, the raw sequencing data was then mapped to the Pf3D7 (*P. falciparum*) reference (v3) genome using *bwa-mem* software (default parameters for Illumina data)^48^. SNPs and short insertions and deletions (indels) were called using the *samtools*, freebayes, and GATK software suites^49–51^. The minimum base quality for gatk and freebayes was changed to 30 rather than their default parameters of 10 and 0, respectively. SNPs occurring in low quality or low coverage regions were discarded. Mixed call SNPs were assigned genotypes determined by a ratio of coverage in which nucleotide calls were 80% or higher. SNPs were annotated using *bcftools* consequence calling, which predicts functional variant consequences^49,52^.

### Ethical approval and consent to participate

All experimental protocols and research were performed in accordance with relevant guidelines and regulations. Research was approved by the Mount Kenya University Independent Ethics Research Committee (MKU-IERC) (Approval reference: P609/10/2014) and the Ethics Committee at Osaka Metropolitan University (Approval number: 3206). Workshops and sensitisation meetings were carried out with communities to attain community consent to study participation. Written informed consent was obtained from all study participants whose parasite DNA was used in this study.

## Supplementary Information

Supplementary Information.

## Data Availability

All raw sequence data is available from the ENA (project accession number PRJEB58092).
