# Supplementary material for: Drug resistance profiling of asymptomatic and low-density Plasmodium falciparum malaria infections on Ngodhe island, Kenya, using custom dual-indexing next-generation sequencing
Source: Sci Rep. 2023 Jul 14;13:11416. doi: 10.1038/s41598-023-38481-3 (PMC10349106; doi:10.1038/s41598-023-38481-3)
Supplement: Supplementary file 1 — Supplementary Information. [file 41598_2023_38481_MOESM1_ESM.pdf]

**Figure S1: Flow chart describing the bioinformatic pipeline of analysis from raw sequencing reads to variant calling.**

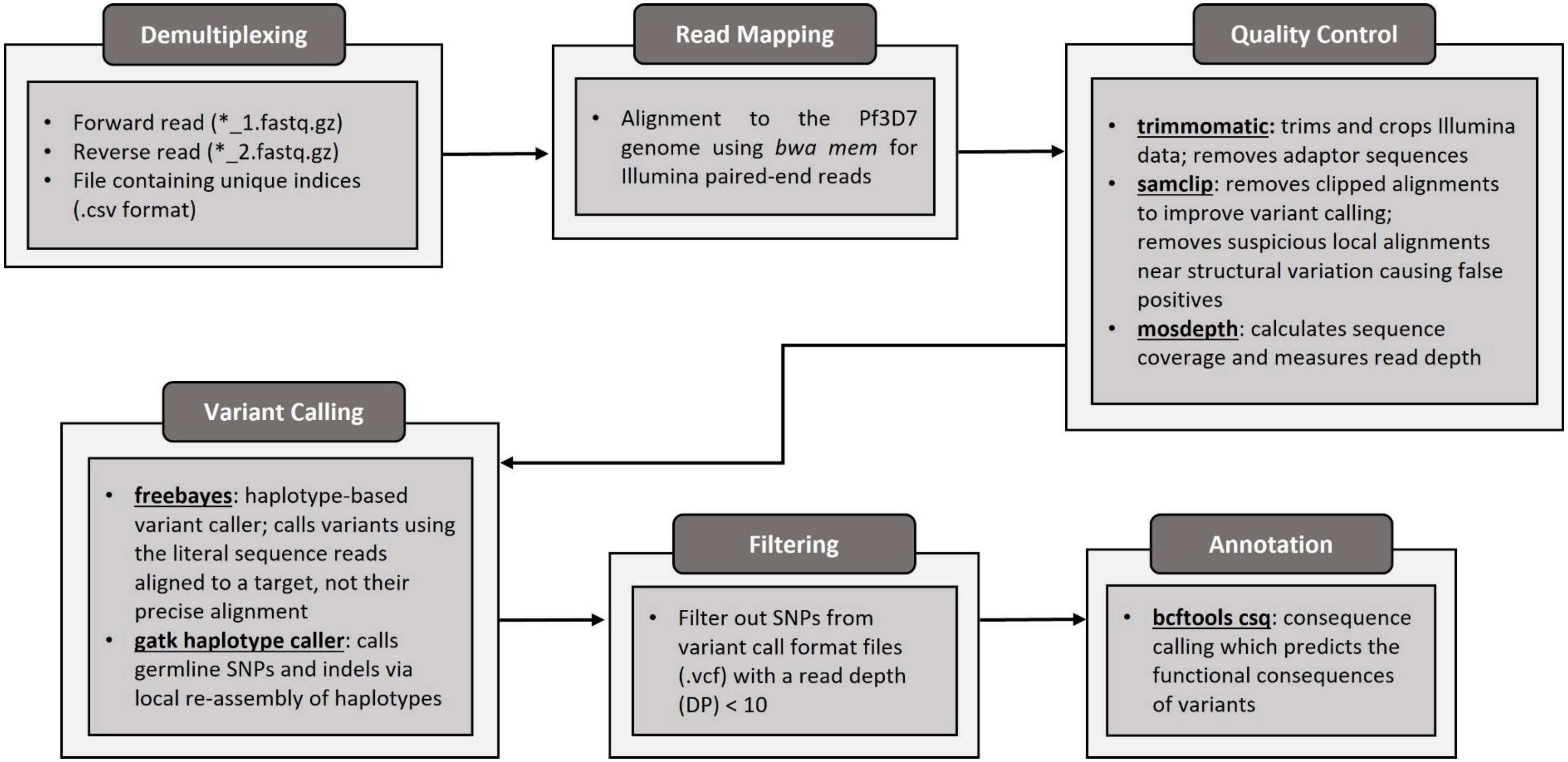

**Table S1: Read depth of sequencing reads across the 5 sequenced resistance genes (*n* = 70).**

| Gene | Median | Max |
|---|---|---|
| *Pfcrt* | 155 | 471 |
| *Pfmdr1* | 69 | 3962 |
| *Pfdhps* | 103 | 2793 |
| *Pfdhfr* | 126 | 2604 |
| *Pfk13* | 1760 | 31280 |

**Table S2: Primers used for the amplification of amplicons on *Pfcrt, Pfmdr1, Pfdhps, Pfdhfr,* and *Pfk13*. (PCR reaction; S = simplex reaction, M = Multiplex reaction).**

| Amplicon name | Gene ID | Primer Sequence | PCR reaction |
|---|---|---|---|
| *Pfcrt* | PF3D7_0709000 | Forward: TCTTGTCTTGGTAAATGTGCTCA | S1 |
| | | Reverse: AGGCCAAAATGACTGAACAGG | |
| *Pfmdr1.1* | PF3D7_0523000 | Forward: CGTTTAAATGTTTACCTGCACAAC | M1 |
| | | Reverse: TGACACCACAAACATAAATTAACGG | |
| *Pfmdr1.7* | | Forward: TTTGTCCAATTGTTGCAGCTGTATTAACTTT | M4 |
| | | Reverse: TGCATTTTCTGAATCTCCTTTTAAGGACATT | |
| *Pfmdr1.8* | | Forward: GGTAAAGTTGATATTAAAGATGTAAATTTCC | M3 |
| | | Reverse: TGGTCCAACATTTGTATCATATTTATTTGG | |
| *Pfdhfr* | PF3D7_0417200 | Forward: ATGATGGAACAAGTCTGCGACGTTTTCGA | M2 |
| | | Reverse: CTAAAAATTCTTGATAAACAACGGAACCTCC | |
| *Pfdhps.3* | PF3D7_0810800 | Forward: ACCATCAGATGTTTATATAACAAATATGTG | M3 |
| | | Reverse: CTGGATTATTTGTACAAGCACTAATATCA | |
| *Pfdhps.4* | | Forward: AGAATGTGTTGATAATGATTTAGTTGATAT | M4 |
| | | Reverse: GATATAAAAGTTGATCCTTGTCTTTCCT | |
| *PfK13.4* | PF3D7_1343700 | Forward: TAAGTGGAAGACATCATGTAACCAGAGA | M2 |
| | | Reverse: CTTCTACATTCGGTATAATAGAAGAGCC | |
| *PfK13.5* | | Forward: ATGATGGCTCTTCTATTATACCGAATG | M1 |
| | | Reverse: GCTATTAAAACGGAGTGACCAAATCTG | |
